# External treatment of traditional Chinese medicine for radiation enteritis: A protocol for systematic review and meta-analysis

**DOI:** 10.1097/MD.0000000000026014

**Published:** 2021-05-28

**Authors:** Hui Luo, Yanling Chen, Yian Zhang, Yufei Wang, Hualan Deng, Dejiao Yao

**Affiliations:** a Clinical Medical College, Chengdu University of Traditional Chinese Medicine, Chengdu; b The First Clinical College, Guangzhou University of Chinese Medicine, Guangzhou; c The Affiliated Hospital of Chengdu University of Traditional Chinese Medicine, Chengdu, China.

**Keywords:** radiation enteritis, external treatment, traditional Chinese medicine, meta-analysis

## Abstract

**Background:**

Radiation enteritis (RE) is a common complication that often occurs after radiotherapy for abdominal and pelvic malignancies. RE could influence patients’ quality of life seriously and it is difficult to cure by conventional treatments. A lot of studies have revealed that the external treatment of traditional Chinese medicine (TCM) for RE is a safe and economical approach, but there is no relevant systematic review. The present study performed a systematic review and meta-analysis to compare TCM external treatment and conventional treatment for RE to evaluate the effectiveness and safety of external treatment of traditional Chinese medicine in the treatment of RE.

**Methods:**

Cochrane Library, PubMed, Embase, China National Knowledge Infrastructure (CNKI), Wan-Fang database, VIP Chinese Science and Technique Journals Database, and the Chinese Biomedical Literature Database (CBM) were searched. The time of publication was limited from inception to April, 2021. Two reviewers independently searched for the selected articles and extract the data. The RevMan V.5.3 statistical software (Cochrane Collaboration) and Stata V.16.0 software were used to conduct the meta-analysis.

**Results:**

We will show the results of this study in a peer-reviewed journal.

**Conclusion:**

This meta-analysis will provide reliable evidence for external treatment of TCM in the treatment of RE.

**INPLASY registration number:**

INPLASY202140120

## 1 Introduction

Radiation enteritis (RE) often refers to a common complication of abdominal and pelvic malignant tumors after radiotherapy. In clinical work, the course of acute radiation enteritis is generally limited to no >3 months, and those more than 3 months or up to several years are called chronic radiation enteritis.^[1]^ A team has reported that about 70% of cancer patients need to receive radiotherapy. In this part of patients receiving abdominal and pelvic radiotherapy, about 50% to 70% suffer from acute radiation enteritis, and 5% to 11% will develop into chronic radiation enteritis.^[2]^ The main symptoms of RE were abdominal pain, diarrhea, bloody mucus, incontinence, nausea, and vomiting.^[3–5]^ Anti-inflammatory, hemostatic, analgesic, and antidiarrheal methods are often used in the treatment of RE.^[6,7]^ Although the use of above conventional treatment has achieved good results, but many patients’ symptoms are only temporarily relieved, and is easy to relapse.^[8]^ Therefore, this disease is difficult to cure. As a result, many scholars have attempted to identify more treatment methods, and the external treatment of traditional Chinese medicine (TCM) was identified as a more safe and effective approach. A large number of clinical studies on the treatment of RE with external treatment of TCM have been reported, but there is no relevant systematic review. Hence this study aims to comprehensively and systematically evaluate the efficacy and safety of external treatment of TCM in the treatment of RE.

## 2 Methods and analysis

The study will follow the Preferred Reporting Items for Systematic Review and Meta-Analysis (PRISMA) 2015 statement.^[9,10]^ Enrollment was completed in April 2021 on the International Platform of Registered Systematic Review and Meta-Analysis Protocols (INPLASY), with registration number: INPLASY202140120. The platform can be accessed at https://inplasy.com/inplasy-2021-4-0120/. This study did not require ethical approval because it did not involve participants’ information or violation of their privacy.

### 2.1 Research criteria

The inclusion criteria will be as follows:

1. Randomized controlled trials;
2. The subjects were patients with radiation enteritis, and there were specific diagnostic criteria;
3. The experimental group was treated with external treatment of traditional Chinese medicine (including enema of traditional Chinese medicine, acupuncture and moxibustion, ear point pressing beans, and so on) or combined with conventional treatment, whereas the control group was only treated with conventional treatment;
4. At least one of the following outcome indicators should be included: efficacy rate, TCM symptom score, Karnorfsky performance scale, inflammatory cytokine level, and so on.

In contrast, the exclusion criteria will be as follows:

1. Nonrandomized controlled studies;
2. Summary, treatment experience, and zoopery;
3. The experimental group and the control group were both treated with traditional Chinese medicine.
4. Data cannot be extracted and the quality of the literature is poor or repeated.

The main result will include efficacy rate.

The secondary outcomes will be the adverse events, Karnorfsky performance scale score, inflammatory cytokine level, and TCM symptom score. The TCM symptom score includes abdominal pain score, diarrhea score, mucus purulent stool score, and so on.

### 2.2 Search strategy

A systematic search of Cochrane Library, PubMed, Embase, China National Knowledge Infrastructure (CNKI), Wan-Fang database, VIP Chinese Science and Technique Journals Database, and the Chinese Biomedical Literature Database (CBM) will be performed, reviewing publications from the beginning of the database to April 2021. The languages are limited to English and Chinese. The main search terms are as follows: radiation enteritis, external treatment of traditional Chinese medicine, retention enema, acupuncture and moxibustion, ear point pressing bean, acupoint application, acupoint injection, and so on. The subject word and free word retrieval will be combined during search. Grey literature will also be searched to avoid literature omission. The search strategy for PubMed is shown in Table 1.

**Table 1:** Search strategy used in the PubMed database.

Number	Search terms
1	Radiation enteritis
2	Radioactive enteritis
3	Radiation colitis
4	Radiation proctitis
5	Or/1–4
6	Randomized controlled trials
7	Controlled clinical trial
8	Clinical trials
9	Randomized
10	Placebo
11	Or/6–10
12	Traditional Chinese medicine
13	External treatment
14	Retention enema
15	Acupuncture
16	Moxibustion
17	Ear point pressing bean
18	Acupoint application
19	Acupoint injection
20	Or/12–19
21	5, 11, and 20

### 2.3 Data collection and analysis

#### 2.3.1 Literature screening

**Figure 1:**
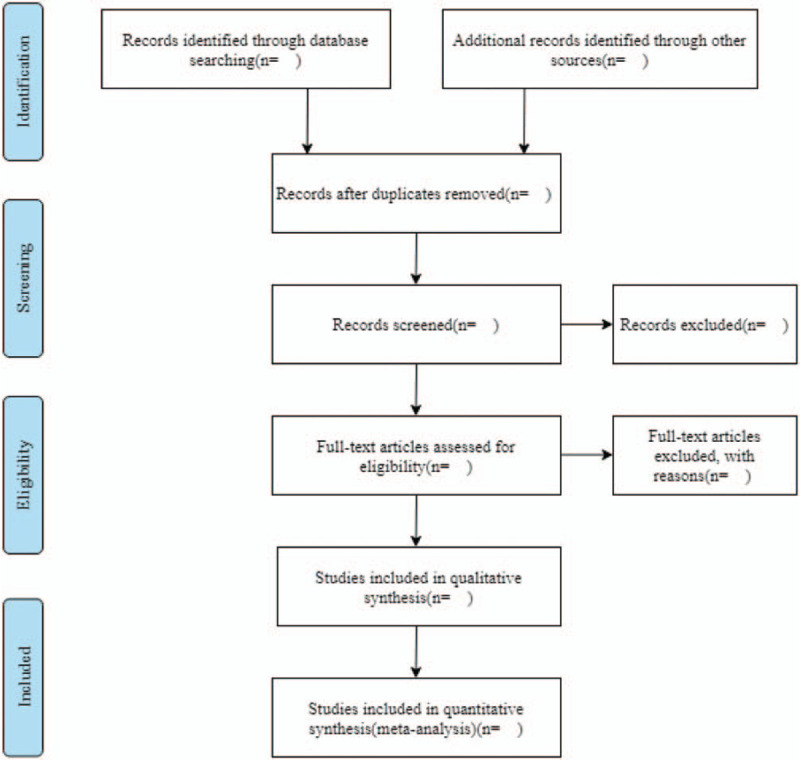
Flowchart of literature selection.

During the first screening, 2 researchers will look through the title of the literature and its abstract, excluding those that obviously do not meet the criteria. Secondary screening will involve full-text reading of potentially qualified literature to further exclude unqualified literature. A third screening will be performed if the information in the literature cannot be clearly included, which will hence involve contacting the authors by mail, and other means to obtain relevant information. The details of the study selection and identification process will be presented in a flowchart (Fig. 1).

#### 2.3.2 Literature evaluation

The methodological quality of randomized controlled trials was evaluated by using the Cochrane Collaboration's risk of bias tool. This tool focused on the following domains: generation of random sequence, allocation concealment, blinding of participants and personnel, incomplete outcome data, duration of follow-up, selective reporting, and other bias. Any disagreements between the reviewers were resolved through discussion, or by seeking advice from a third reviewer. The quality of the evidence was evaluated using the Grading of Recommendations Assessment, Development and Evaluation (GRADE), which determines the quality of the evidence according to the primary outcomes, consistency of the results, imprecision, and publication bias. The strength of the evidence was graded as high, medium, low, and very low quality.

#### 2.3.3 Statistical analysis

When a meta-analysis was possible, the data synthesis was presented using the RevMan V.5.3 statistical software. The statistically data was meaningful when *P* < .05. The mean difference with 95% confidence interval was used to evaluate the continuous outcomes, and the relative risk (RR) with 95% confidence interval was used to evaluate the dichotomous data. The fixed effects model (*I*^2^ < 50%) was used to estimate the RR and mean difference. The random effects model (*I*^2^ > 50%) was used for the synthesis of the data which considered substantial statistical heterogeneity. The funnel plots were indicated for obvious publication bias.

#### 2.3.4 Subgroup analysis

When there is significant heterogeneity in the trials and have enough randomized controlled trials, then we will analyze the subgroups according to age, sex, course of intervention, and type of intervention in the experimental and control groups.

#### 2.3.5 Sensitivity analysis

The sensitivity analysis was conducted to evaluate the robustness and reliability of the pooled results. If adequate data were available for analysis, a sensitivity analysis for the primary outcomes was conducted to test the strength of the review conclusions, which included the quality of the methods and studies, and the impact of the sample size and missing data.

#### 2.3.6 Publication bias analysis

If >10 studies were included, the funnel plot was used to determine the publication bias.^[11,12]^ Egger test and Begg test were performed to quantitatively assess the publication bias using the Stata V.16.0 software. The results were estimated based on the Cochrane Handbook for Systematic Reviews of Interventions.

#### 2.3.7 Ethics and communication

Because this study is a secondary analysis, it will not involve patient and public information, and hence does not require ethical review. The results of this study will be disseminated through peer-reviewed publications, journals, and academic exchanges.

## 3 Discussion

According to some researchers’ reports, in primary and metastatic abdominal and pelvic tumors, radiotherapy has significant effect on improving patients’ overall survival, which also leads to radiation enteritis. In some patients, radiation enteritis may not only aggravate the primary disease, but also cause systemic inflammatory response syndrome, multiple organ dysfunction syndrome, and so on. In these circumstances, RE could endanger the lives of patients, and even some serious radiation enteritis must be treated by surgery. However, it also has a great impact on patients’ quality of life.

Hormone, antibiotic, intestinal mucosal protectants, probiotics, and hyperbaric oxygen are mainly used in the treatment of RE as a conventional treatment currently. However, the above-mentioned therapies have some disadvantages, such as large side effects, expensive price, and different curative effects. In recent years, increasing clinical reports have revealed that external treatment of TCM could achieve certain efficacy in the treatment of RE.^[13,14]^ However, there is no comparison on the external treatment of TCM for the direct or indirect treatment of RE. Therefore, the present study conducted a meta-analysis to directly or indirectly compare TCM external treatment to conventional treatments for RE, to determine which therapeutic measure has the best relative efficacy and safety, and provide the best evidence for clinical practice. In future research, we should conduct multicenter, large-sample and high-quality researches, to establish a more objective and systematic treatment.

## Author contributions

**Conceptualization:** Yufei Wang, Hui Luo.

**Methodology:** Yufei Wang, Hui Luo, Yanling Chen.

**Administration:** Hui Luo.

**Resources:** Yanling Chen, Yian Zhang, Hualan Deng.

**Validation:** Yufei Wang, Dejiao Yao.

**Software:** Yanling Chen, Yian Zhang.

**Supervision:** Dejiao Yao.

**Writing – original draft:** Yufei Wang, Hui Luo.

**Writing – review & editing:** Hualan Deng.

**Conceptualization:** Hui Luo, Yufei Wang.

**Data curation:** Hui Luo.

**Formal analysis:** Hui Luo, Yufei Wang.

**Funding acquisition:** Dejiao Yao.

**Investigation:** Hui Luo, Hualan Deng.

**Methodology:** Hui Luo, Yufei Wang, Yanling Chen.

**Project administration:** Hui Luo.

**Resources:** Yanling Chen, Yian Zhang, Hualan Deng.

**Software:** Yanling Chen, Yian Zhang.

**Supervision:** Dejiao Yao.

**Validation:** Hui Luo, Yufei Wang, Dejiao Yao.

**Visualization:** Hui Luo, Yanling Chen.

**Writing – original draft:** Hui Luo, Yufei Wang.

**Writing – review & editing:** Hualan Deng.

## References

[1] Ding X Li QQ Li P. Fecal microbiota transplantation: a promising treatment for radiation enteritis? Radiother Oncol 2020;143:12–8. 32044171 10.1016/j.radonc.2020.01.011

[2] Ashburn JH Kalady MF. Radiation-induced problems in colorectal surgery. Clin Colon Rectal Surg 2016;29:85–91. 27247532 10.1055/s-0036-1580632 PMC4882181

[3] Membrive Conejo I Reig Castillejo A Rodríguez de Dios N. Prevention of acute radiation enteritis: efficacy and tolerance of glutamine. Clin Transl Oncol 2011;13:760–3. 21975340 10.1007/s12094-011-0729-3

[4] Vidal-Casariego A Calleja-Fernández A de Urbina-González JJO. Efficacy of glutamine in the prevention of acute radiation enteritis: a randomized controlled trial. JPEN J Parenter Enteral Nutr 2014;38:205–13. 23471208 10.1177/0148607113478191

[5] Vidal-Casariego A Calleja-Fernández A Cano-Rodríguez I. Effects of oral glutamine during abdominal radiotherapy on chronic radiation enteritis: a randomized controlled trial. Nutrition 2015;31:200–4. 25466666 10.1016/j.nut.2014.08.003

[6] Chen L Zhou L Yao SQ. Therapeutic observation of Huangteng Mixture on acute radiation enteritis with accumulated dampness heat. Chinese Traditional Patent Medicine 2016;38:2556–60.

[7] Zhu LW Wang SY Li YL. Clinical observation on integrated traditional Chinese and Western medicine on radiation enteritis. CJGMCM 2018;33:1942–4.

[8] Paquette IM Vogel JD Abbas MA. The American society of colon and rectal surgeons clinical practice guidelines for the treatment of chronic radiation proctitis. Dis Colon Rectum 2018;61:1135–40. 30192320 10.1097/DCR.0000000000001209

[9] Feigin VL Roth GA Naghavi M. Global burden of stroke and risk factors in 188 countries, during 1990-2013: a systematic analysis for the Global Burden of Disease Study 2013. Lancet Neurol 2016;15:913–24. 27291521 10.1016/S1474-4422(16)30073-4

[10] Forouzanfar MH Alexander L Anderson HR. Global, regional, and national comparative risk assessment of 79 behavioural, environmental and occupational, and metabolic risks or clusters of risks in 188 countries, 1990-2013: a systematic analysis for the Global Burden of Disease Study 2013. Lancet (London, England) 2015;386:2287–323. 10.1016/S0140-6736(15)00128-2 PMC4685753 26364544

[11] Sutton AJ Duval SJ Tweedie RL. Empirical assessment of effect of publication bias on meta-analyses. BMJ 2000;320:1574–7. 10845965 10.1136/bmj.320.7249.1574 PMC27401

[12] Egger M Davey Smith G Schneider M. Bias in meta-analysis detected by a simple, graphical test. BMJ 1997;315:629–34. 9310563 10.1136/bmj.315.7109.629 PMC2127453

[13] Wang L Na JW Dong XH. Clinical efficacy and nursing experience of retention enema with chinese herbal medicine on acute radiation enteritis. JETCM 2018;27:608–11.

[14] He XY Gao ZY Sun YC. Clinical efficacy of anchang formula for treating acute radiation enteritis and its effects on serum expressions of IL-2 IFN-, and IL-10. CJITWM 2018;38:795–8.

